# Pressure Dependent Product Formation in the Photochemically Initiated Allyl + Allyl Reaction

**DOI:** 10.3390/molecules181113608

**Published:** 2013-11-04

**Authors:** Lars Seidel, Karlheinz Hoyermann, Fabian Mauß, Jörg Nothdurft, Thomas Zeuch

**Affiliations:** 1 Lehrstuhl Thermodynamik/Thermische Verfahrenstechnik, BrandenburgischeTechnische-Universität, Siemens-Halske-Ring 8, Cottbus D-03046, Germany; E-Mails: Lars.Seidel@tdtvt.de (L.S.); Fabian.Mauss@tdtvt.de (F.M.); 2 Institut für Physikalische Chemie, Georg-August-Universität, Tammannstr. 6, Göttingen D-37077, Germany; E-Mails: khoyerm@gwdg.de (K.H.); jnothdu@gwdg.de (J.N.)

**Keywords:** allyl radical, allyl self-reaction, aerosol precursor chemistry

## Abstract

Photochemically driven reactions involving unsaturated radicals produce a thick global layer of organic haze on Titan, Saturn’s largest moon. The allyl radical self-reaction is an example for this type of chemistry and was examined at room temperature from an experimental and kinetic modelling perspective. The experiments were performed in a static reactor with a volume of 5 L under wall free conditions. The allyl radicals were produced from laser flash photolysis of three different precursors allyl bromide (C_3_H_5_Br), allyl chloride (C_3_H_5_Cl), and 1,5-hexadiene (CH_2_CH(CH_2_)_2_CHCH_2_) at 193 nm. Stable products were identified by their characteristic vibrational modes and quantified using FTIR spectroscopy. In addition to the (re-) combination pathway C_3_H_5_+C_3_H_5_ → C_6_H_10_ we found at low pressures around 1 mbar the highest final product yields for allene and propene for the precursor C_3_H_5_Br. A kinetic analysis indicates that the end product formation is influenced by specific reaction kinetics of photochemically activated allyl radicals. Above 10 mbar the (re-) combination pathway becomes dominant. These findings exemplify the specificities of reaction kinetics involving chemically activated species, which for certain conditions cannot be simply deduced from combustion kinetics or atmospheric chemistry on Earth.

## 1. Introduction

The allyl radical (C_3_H_5_) is the smallest π-conjugated radical. Its reactions with other hydrocarbon radicals are a building block for basic kinetic schemes for hydrocarbon combustion and for photochemical models of the planetary atmospheres [1,2,3,4]. Due to its exceptional stability it is often present in significant concentrations in flames and during pyrolysis processes [5,6,7,8,9,10,11]. Modeling studies show that the knowledge of accurate kinetic data for the reactions of unsaturated C_3_-species is essential for the description of reactive particle formation processes [4,9,10,12,13]. 

For assessing the role of the allyl chemistry in complex reactive systems it is crucial to characterize not only the rates of allyl-consuming reactions, but also the product branching in the respective pathways and its pressure dependency. Both the formation of secondary organic aerosols on Earth and the formation of organic haze on Titan were shown to be pressure dependent in laboratory experiments [14,15]. 

For the reaction of allyl radicals with oxygen atoms the significance of reaction channels via C-C fission was established in the past [10,16,17]. In the present study, we have applied our technique of laser flash photolysis combined with the quantitative detection of reactants and products by FTIR spectroscopy [10,18,19] to the study of the allyl radical self-reaction. Here, the competition between recombination (more correctly combination, however, in the literature recombination is the typical designation) and disproportionation is the focus and the potential influence of radical forming channels as recently reported for the vinyl radical self- reaction [20] at low pressures.

In many earlier studies [21,22,23] the rate of the allyl self-reaction was determined experimentally and recently also theoretically [23,24]. The product formation, however, received less attention until recently. The recombination reaction (1) was found to be the only significant channel going back to an experimental study by James and Kambanis, who reported the disproportionation route yield to be below 1% [25]. This finding was confirmed in recent work including one study with direct product detection at low pressure [26,27,28]. The knowledge about the branching between recombination and disproportionation is important with regard to benzene formation in flames, where many allyl reactions are involved in considered benzene formation pathways [7,9,10,29]. In addition pressure effects have to be taken into account since the nascent allyl radicals, formed by flash photolysis, and hence also the recombination product may be energized and show different reaction pathways at low and high pressures. The two known pathways for the allyl + allyl reaction are listed below:

C_3_H_5_ + C_3_H_5_→C_6_H_10_ (1)

C_3_H_5_ + C_3_H_5_→H_2_CCCH_2_ + C_3_H_6_ (2)

Due to chemical activation of 1,5-hexadienyl formed from activated allyl radicals the reaction pathway (2) may be alternatively represented by the following scheme:

C_3_H_5_^*^ + C_3_H_5_^*^→[C_3_H_5_-C_3_H_5_]^*^→H_2_CCCH_2_ + C_3_H_6_ (3)

The excited transition state indicates in anticipation of our results that the fraction of the disproportionation route may depend on the internal energy of allyl radicals at low pressures. 

The objectives of the present study are: (i) the experimental identification of reaction channels in the allyl self-reaction kinetics and the assessment of their relative branching fractions as a function of pressure; (ii) the rationalization of the mechanisms that operate in this type of laser flash photolysis experiments by means of kinetic modeling.

## 2. Results and Discussion

### Product Branching

For studying the formation of the stable products, the allyl radicals were generated by photodecomposition of the precursors allyl chloride, allyl bromide, or 1,5-hexadiene. The pros and cons of the different precursors have been discussed in a previous paper in the context of the allyl + oxygen atom reaction [10] and they are also considered below in the context of the allyl self-reaction. Product spectra were taken in intervals of 50–100 laser shots each up to a total number of 200–400 laser shots. The scanning time for the IR measurements is typically 10 to 20 s (100 scans). The number of laser shots was chosen such that a total precursor conversion of about 10%–15% was achieved. In order to avoid secondary reactions this upper limit was not exceeded in all experiments. The effect of variation of initial precursor concentrations was tested but no measurable influence on the observed product branching was observed.

The IR spectra were analyzed in the spectral range of 3800–600 cm^−1^ by comparing the product spectra with spectra of the pure substances. For product quantification calibration curves were recorded and determined for all identified compounds at the same temperature 296 ± 1 K and in the pressure range of 0.4–500 mbar. Due to the low molecular mass and the different functional groups (-C-C-, -C≡C-, -C=C-) absorbing in different regions of the IR spectrum, an unambiguous and quantitative calibration of most compounds was possible.

**Figure 1:**
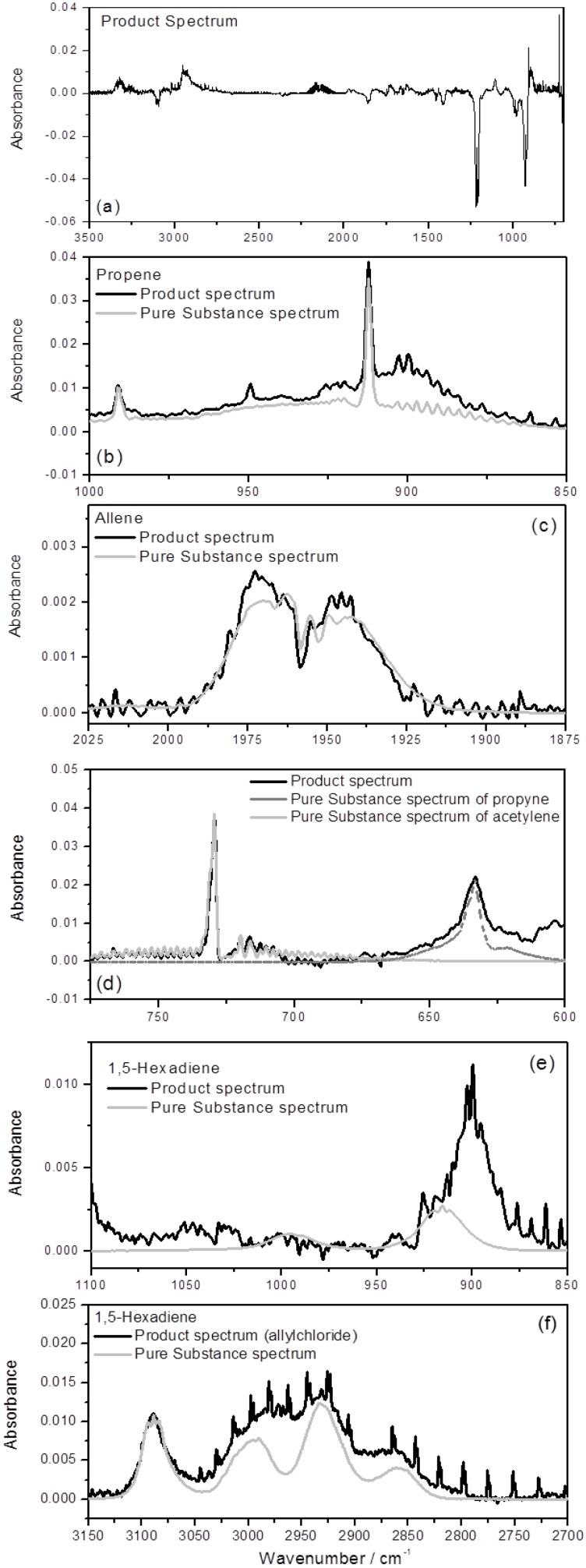
Product formation in the C_3_H_5_ + C_3_H_5_ reaction system with C_3_H_5_Br as precursor: identification of major products, (**a**) complete product spectrum, raw data (**b**) propene, (**c**) allene, (**d**) propyne and acetylene; (**e**) upper limit for 1,5-hexadiene; p(C_3_H_5_Br) = 0.3 mbar, (**f**) detection of CH_2_CH(CH_2_)_2_CHCH_2_ from precursor C_3_H_5_Cl, p(C_3_H_5_Cl) = 0.3 mbar; p(total) = 4 mbar, bath gas Ar, T = 296 ± 1 K.

Examples for unequivocal product identification at 4 mbar are given in Figure 1 for the precursor allyl bromide. Here, separate regions of the IR spectra reveal the unambiguous identification of the following stable products: propene (C_3_H_6_), allene (H_2_CCCH_2_), propyne (HCCCH_3_), and acetylene (C_2_H_2_). We note that 1,5-hexadiene can only be a minor product at 4 mbar for this precursor (see panel (e) of Figure 1). The significant formation of propyne and acetylene is surprising because neither reaction (1) nor (2) produce these species. In addition, the high yield of allene when assigned to reaction (2) is in contrast to previous work [25,26,27,28]. We note that there are species which could not be detected by IR like H_2_ or which were not identified. However, the carbon mass recoveries for the precursors allyl bromide (85%) and 1,5-hexadiene (95%–100%) indicate that unidentified products come from minor channels. In case of allyl chloride the carbon mass recovery was lower and most of the products, which have not been detected, are Cl substituted species, which belong to the unavoidable Cl atom reaction with the double bond of allyl chloride (see also the Experimental section below).

**Figure 2:**
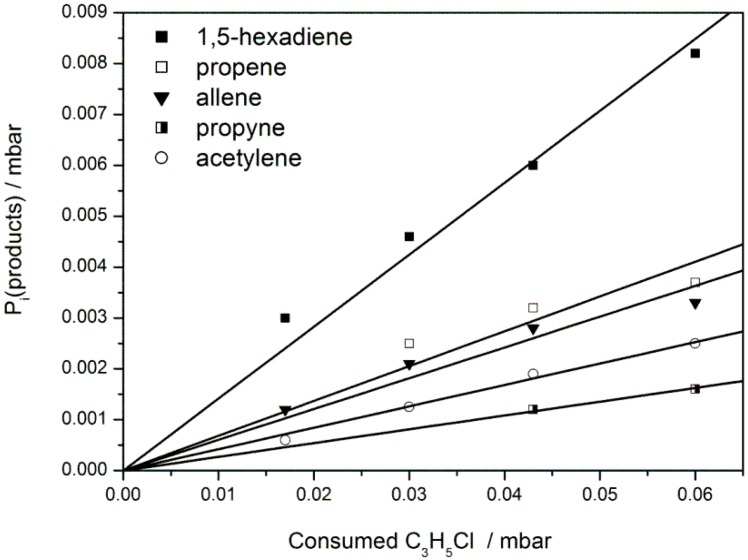
The C_3_H_5_ + C_3_H_5_ reaction: formation of products *vs.* consumption of the precursor C_3_H_5_Cl, p(C_3_H_5_Cl) = 0.3 mbar; p(total) = 4 mbar, bath gas Ar, T = 296 ± 1 K.

It has been demonstrated in previous studies on hydrocarbon radical reactions with O atoms that the influence of the secondary chemistry can be largely suppressed when high initial radical concentrations are generated in a nanosecond time window by laser photolysis [7,17]. The key point is that the consumption of non-radical primary products, formed in the microsecond time window of the main reaction, and of molecular photolysis products by secondary chemistry is suppressed in this type of experiment with its specific chemistry. Instead, the secondary chemistry is dominated by the faster radical/radical or radical/atom reactions [10,18]. This assumption was validated by kinetic modeling for a specific class of reactions and precursors [18]. In the present study the high product yields of propene, propyne, and allene is an intriguing and unexpected result and the former assumption has to be put to the test. To this end, the use of three different precursors with specific features of their side chemistry as well as the variation of the pressure should provide constraints for plausible mechanistic interpretations. In Table 1 are shown final product yields of propene, propyne, allene, acetylene, and 1,5-hexadiene at 4 mbar in the experiments via the precursors allyl bromide, allyl chloride and 1,5-hexadiene. Figure 2 illustrates the averaging of the product yields by analyzing the product formation at different precursor conversions for the precursor allyl chloride. The yields are given as a function of the precursor consumption; the errors correspond to the estimated errors due to the calibration of product concentration based on their specific IR absorption bands. This error is typically derived from residual absorption features after subtraction of pure substance spectra from the IR product spectrum. For many of the species found in this work the calibration spectra derived in previous work could be used [10,19]. For the larger product 1,5-hexadiene potential spectral interferences with other reaction products implied a higher estimated error compared to the smaller products (see Figure 1e). This uncertainty only applies for the precursor allyl bromide in the low pressure experiments, for which we can only give an upper limit for the channel fraction of 1,5-hexediene (see Table 1). At high pressure and using allyl chloride the assignment is much clearer (see Figure 1f). In [18] a more detailed discussion of the assessment of errors was presented. 

**Table 1:** Experimentally determined final product yields relative to precursor consumption for the C_3_H_5_ + C_3_H_5_ reaction for different precursors. p(C_3_H_5_Br) = 0.3 mbar; p(C_3_H_5_Cl) = 0.3 mbar; p(CH_2_CH(CH_2_)_2_CHCH_2_) = 0.4 mbar. p(total) = 4 mbar, bath gas Ar, T = 298 K.

Precursor Product	C_3_H_5_Br	C_3_H_5_Cl	1,5-hexadiene
Propene	30 ± 5%	7 ± 2%	80 ± 10%
Propyne	10 ± 3%	3 ± 1%	24 ± 5%
Allene	6 ± 2%	6 ± l%	25 ± 5%
Acetylene	15 ± 3%	4 ± 1%	26 ± 4%
1,5-Hexadiene	<5%	14 ± 5%	—

As stated above, at low pressure (4 mbar) surprisingly high yields of propyne and acetylene but also propene and allene were found for all precursors (see Table 1). Therefore, the photolysis induced reaction kinetics are seemingly coupled to photochemistry and secondary chemistry that must be considered for the rationalization of end product formation. We start the discussion with a comparison of the present results to those of a previous study using the precursor 1,5-hexadiene.

For the 1,5-hexadiene photolysis, Pilling and co-workers [21] reported two allyl radicals (68%) as the major photolysis products at 193 nm, the formation of propene and allene was identified as the only significant molecular channel with a fraction of 27%. This finding was confirmed recently by Selby *et al.* [26] reporting 26% for this channel using the propene concentration for calibration. We find a fraction of (25 ± 3%) for this molecular channel based on propene yields relative to consumed 1,5-hexadiene in experiments in which allyl radicals were scavenged by O atoms in the co-photolysis with SO_2_ [10]. The formation of propyne was not reported, but no comment was given whether allene/propyne could be distinguished in their analysis, which is not trivial using GC [30]. We note that single photon ionization and IR spectroscopy allow their distinction on the basis of the slightly different ionization energies of the two C_3_H_4_ species [31] and their characteristic vibrational modes (see Figure 1), respectively. The existence of minor radical producing channels, like CH_3_ + C_5_H_7_ was deduced indirectly by Tulloch *et al.* [21] from characteristic combination products like 1-C_4_H_8_ from the C_3_H_5_ + CH_3_ reaction and observed directly by Selby *et al.* [26]. However, the potential disproportionation reaction of two allyl radicals and radical forming channels were not taken into account by Pilling and co-workers [21]. This part of their work was focused on establishing 1,5-hexadiene photolysis at 193 nm as a clean source of allyl radicals for studying the rate coefficient of the allyl self-reaction.

We now discuss the allyl chloride photolysis. For this precursor comparatively large fractions of final products were found which potentially belong to the disproportionation route or to yet unknown chemistry in case of propyne and acetylene (see Table 1). It is also obvious from Table 1 that the relative yields vary with the precursor indicating precursor specific effects. In the case of allyl chloride, fast reacting Cl atoms are formed during the photolysis at 193 nm. The competitive reactions of Cl atoms with the precursor, olefinic primary products [32,33,34] and, of course, unsaturated radicals [35] may complicate the stable product analysis as we pointed out previously [10]. Assuming kinetics for the reaction allyl + Cl similar to vinyl + Cl [35], this reaction is roughly two times faster than that of the title reaction [21,23]; Selby *et al.* estimate this ratio up to a factor of six in favor of allyl + Cl [26]. As a consequence, an enhanced formation of the abstraction product C_3_H_4_ compared to C_3_H_6_ is expected [35], which was observed in the experiments. The significant dominance of allene over propyne formation in the allyl chloride photolysis experiments suggests that allene is the abstraction product in the allyl + Cl reaction. The comparatively large product yield of 1,5-hexadiene illustrates that allyl radicals are efficiently produced. In total, about 30% of the consumed C_3_H_5_Cl reacted via channel (1). This finding is consistent with (1) being the dominant route at 4 mbar. The possible chemical pathways to propene, propyne, and allene (formed from allyl + Cl) is discussed below in detail for the case of allyl bromide photolysis. 

Allyl bromide photolysis produces allyl radicals and bromine atoms whose reactions are comparatively slow and do not interfere with the fast radical-radical chemistry [36,37,38]. The only fast reaction here is the re-formation of allyl bromide, which was analyzed in detail by Bedjanian *et al.* [39]. They did not observe the alternative H atom abstraction channel being discussed above for vinyl + Cl and allyl + Cl reflecting the different reactivity of bromine and chlorine atoms. In their work, the experiments were conducted at room temperature, which is similar to the present study. Based on these considerations allyl bromide photolysis seems to be a suitable precursor for studying product formation in the allyl self-reaction. The same precursor was applied by Selby *et al.* using flash photolysis at 248 nm [26]. 

The end product analysis for the laser photolysis of allyl bromide at 4 mbar shows that propene is the major product also for this precursor. Different to allyl chloride photolysis significantly less allene than propyne was formed. This seems to bolster a bit the explanation of the enhanced allene formation by the allyl + chlorine atom reaction. We note that for allyl bromide and allyl chloride allene formation is suppressed close to the detection limit in the co-photolysis with sulfur dioxide (SO_2_) at 4 mbar, where oxygen atoms were formed in excess over allyl radicals [10]. This finding excludes the possibility that allene is formed from precursor photolysis, which is a central observation with regard to the interpretation of the results of this study.

We further found that less 1,5-hexadiene was formed in the allyl bromide experiments compared to allyl chloride. In the introduction we discussed that chemical activation may enhance allene and propene formation (channel (3)). Nascent allyl radicals being formed from the decomposition allyl bromide are expected to carry significantly more excess energy compared to their generation from allyl chloride since the energy of the C-Cl bond is about 50 kJ/mol higher than the C-Br bond [40]. Therefore, the increased fraction of channel (1) may be the effect of decreased excess energy of the allyl radical produced from allyl chloride photolysis, which is carried through to the recombination product. 

**Figure 3:**
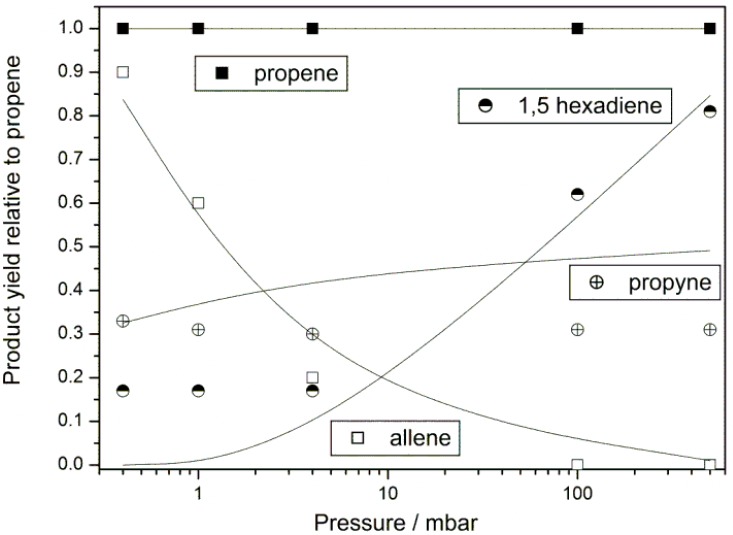
Pressure dependent formation of the products 1,5-hexadiene, allene, propene and propyne between 0.4 and 500 mbar. Precursor allyl bromide, p(C_3_H_5_Br) = 0.3 mbar, bath gas Ar, T = 296 ± 1 K. Symbols show experimental data points, lines show the result of a kinetic simulation using the mechanism of Table 2. The initial concentration of allyl radicals (1 × 10^−9^ mol/cm^3^) in the simulation was estimated from the photolysis laser fluence and the consumption of allyl bromide per 193 nm excimer laser pulse assuming a quantum yield of 1 for the allyl + bromine atom channel.

To further elucidate the role of chemical activation we analyzed the pressure dependence of product formation for the allyl bromide photolysis. The results are illustrated in Figure 3. We notice that allene together with propene show the highest channel fraction at the lowest pressure of 0.4 mbar and that its formation strongly decreases with increasing pressure; it could not be detected at 100 mbar. A further observation is the increasing product yield of the recombination product with increasing pressure above 10 mbar. These observations indicate that chemical activation indeed strongly affects the product branching in the reaction system prepared by allyl bromide photolysis using 193 nm photons. 

**Table 2:** Kinetic model for the simulation of pressure dependent end product yields, which are shown in Figure 3. Rate constant units are mol/cm^3^s for bimolecular and s^−1^ for unimolecular reactions. C_6_H_10_ represents 1,5-hexadiene. * indicates reactions of chemically activated species. For some reactions pressure dependent rate coefficients are derived by logarithmic pressure interpolation between the given pressure values.

No.	Reaction	A	n	Ea	Pressure [bar] or Ref.
1	C_3_H_5_*→ HCCCH_3_ + H	2.0E02	0.00	0.00	
2	C_3_H_5_ + H→C_3_H_6_	1.0E13	0.00	0.00	
3	C6H10*→C_3_H_5_* + C_3_H_5_*	1.0E05	0.00	0.00	
4	C_3_H_5_* + C_3_H_5_*→C_6_H_10_*	1.0E13	0.00	0.00	
5	C_3_H_5_*→ C_3_H_5_	1.0E02	0.00	0.00	4.0E-4
		4.0E02	0.00	0.00	4.0E-3
		1.1E03	0.00	0.00	5.0E-1
6	C_3_H_5_* + C_3_H_5_*→H + H + C_6_H_8_	2.0E11	0.00	0.00	
7	C_3_H_5_ + C_3_H_5_→C_6_H_10_	1.6E13	0.00	0.00	[26]
8	C_3_H_5_ + C_3_H_5_→H_2_CCCH_2_ + C_3_H_6_	1.5E11	0.00	0.00	
9	C_3_H_5_* + C_3_H_5_*→H_2_CCCH_2_ + C_3_H_6_	1.5E12	0.00	0.00	4.0E-4
		1.5E11	0.00	0.00	4.0E-3
		1.0E06	0.00	0.00	5.0E-1
10	C_6_H_10_ + H→C_3_H_6_ + C_3_H_5_	1.0E12	0.00	0.00	1/10 of [28]

At this point the results of recent studies [26,27,28] performed under different conditions are illuminating. Different to the present work, at both, low pressure (1.3–10 mbar) [26] and at high pressure (1–5 bar) [28] a negligible pressure dependence of product formation was observed. The direct product analysis of Selby *et al.* (298–600 K) strongly suggests that the disproportionation channel (2) is of minor importance (<3%). This finding is bolstered by two very recent studies, where end products formed at higher temperatures (600–1700 K) were analyzed [27,28]. Interestingly, the end product spectrum found between 1,000–1,200 K by Fridlyand *et al.* [28] shows similarities to the present study using the pyrolysis of 1,5-hexadiene and allyl iodide as allyl source: propene, allene and propyne were the major products; ethylene and acetylene were the most prominent minor products. The similar yields for allene, propene, and propyne are most pronounced for the lowest pressures in our experiments using allyl bromide as precursor. This may suggest that similar chemical formation mechanisms operate. One difference is that the energy source in our experiment is internal excess energy, provided by photolysis with energy rich 193 nm photons being slowly dissipated to the bath gas at low pressure. A second difference is that unimolecular decomposition reactions driven by chemical activation dominate in the photolysis experiment. Hence, propyne formation may proceed via:

C_3_H_5_^*^→HCCCH_3_ + H (4)

This channel operates as an elementary unimolecular step under pyrolysis conditions at temperatures above 1000 K (reaction R4 in [28]).The mechanism was studied by Mueller *et al.* [41], who found the formation of propyne and hydrogen atoms from the decomposition of activated allyl radicals as the dominant channel.

The reason for the absence of allene, but not propyne, in the end product IR spectra taken above 10 mbar is not clear. A straight forward explanation would be that for the conditions of our experiment the disproportionation reaction (2) may gain importance below 10 mbar and that propyne is formed directly from allyl decomposition (4) also at higher pressure. The absence of higher mole fractions of allene and propene in the direct measurements of Selby *et al.* [26] would then be related to the different photolysis wavelength of 248 nm and consequently a lesser degree of chemical activation (~120 kJ/mol less excess energy). This explanation is along the lines with the discussion of the higher yield of the recombination product in the allyl chloride experiments, in which allyl radicals with lower internal energy were formed. For the highest pressure (500 mbar) most of the allyl radicals react to the recombination product, which is already the case at 4 mbar using allyl chloride (see Table 1).

The product formation can be qualitatively rationalized by kinetic modelling when we assume similarities with pyrolysis experiments around 1100 K [28]. In Table 2 the simple kinetic model is given. The recombination of hot allyl radicals is assumed to produce activated 1,5-hexadiene which decomposes back to two hot allyl radicals. The equilibrium of activated allyl radicals and 1,5-hexadiene is disturbed by loss of activated allyl radicals via the minor disproportionation route and by collisional deactivation of allyl radicals which is included by pressure dependent pseudo-unimolecular step. The rate for this step should be regarded as a switch, tuning the delicate product branching in the hot radical chemistry, rather than an effective strong collision frequency. Allene formation below 10 mbar is explained by a pressure dependent reaction (3), propyne formation by a prompt, pressure independent decomposition of activated allyl, reaction (4). The kinetic simulations showed that an additional source of H atoms is needed to account for the high final product yields of propene and propyne. Different to the modeling part of the recently reported pyrolysis experiment [28], where a much more complex mechanism was used, we incorporated a single, global reaction step (5) to produce H atoms. A similar step was used by Knyazev *et al.* for the vinyl self- reaction to model a laser photolysis experiment [42] and by us for the allyl self-reaction for modelling fuel rich propene flame chemistry [9]:

C_3_H_5_^*^ + C_3_H_5_^*^→C_6_H_8_ + 2H (5)

The structure of C_6_H_8_ was not specified in [9]. In this work it represents a lumped isomer that collects the carbon mass of minor end products such as acetylene and ethylene. However, this step is assumed to be orders of magnitude slower than the recombination reaction. The model qualitatively explains the trends of pressure dependent end product yields of allene, propene and 1,5-hexadiene (see Figure 3, in order to guide the eye, simulated yields at three pressures (0.4, 4 and 500 mbar) are interpolated by B-splines). We note that the mechanism in Table 2 should be regarded as a tentative, empirical model because the treatment of pressure dependent, photochemical activation driven kinetics is difficult for single chemical steps [43]; here many such steps are involved and some kinetic assumptions may proof implausible by a more detailed analysis. Therefore, we did not try to model acetylene formation, which involves additional uncertain chemical steps. We note that the collision frequencies Z at 0.4 mbar and 1 bar are in the order of 10^6^ and 10^10^ s^−1^, respectively. The strong pressure dependence of end product formation in the range of 0.4 to 10 mbar indicates that the specific rate coefficients for unimolecular steps and effective bimolecular collision frequencies of hot allyl radicals are below 10^6^ to 10^7^ s^−1^. This is the case for the hot species chemistry in our model using the estimated initial allyl radical concentration of 1 × 10^−9^ mol/cm^3^. A more specific discussion including vibrational relaxation is beyond the scope of this work with regard to the largely empirical character of the mechanism; see also the discussion in [18].

The discussion above has essentially demonstrated two important points. Firstly, it is clear that in the framework of the present study we cannot give a detailed mechanistic explanation for the high final product yields of propene, propyne and, at low pressure, allene, and acetylene. Secondly, we see in our experiments an end product spectrum, which is dominated by reaction kinetics of energized species. In our experiment propene, allene, and propyne formation is faded out by the increase of pressure (precursor allyl bromide) or the decrease of allyl excess energy after photolysis (precursor allyl chloride). In the recent pyrolysis induced study of the allyl + allyl reaction by Fridlyand *et al.* the formation of these species is faded out by reducing the temperature [28]. We can definitely exclude that the results of the present work are purely related to precursor induced side reactions. The formation of allene, propyne, and propene is largely suppressed when the allyl + allyl kinetics are turned off by scavenging allyl radicals with O atoms formed from SO_2_ co-photolysis with allyl bromide and allyl chloride under otherwise identical conditions [10]. In case of allyl bromide, ~85% of consumed precursor was reacted to the above-mentioned products; ~30% was converted to propene at 4 mbar. The pronounced pressure dependence of end product formation in the allyl bromide case is absent in combustion related studies of allyl + allyl [28]. The effect is related to the specific experimental conditions in this work, e.g., low pressure, high radical concentrations and high photolysis photon energies. 

Finally, we would like to briefly discuss some aspects of the present results in the broader context of “high-energy” chemistry. Using even higher photon energies [44,45] or electrons as energy source [14,45,46], complex organic compounds can be generated from small precursor species such as CH_4_ and N_2_. These experiments aim at simulating the complex photochemistry of planetary atmospheres in laboratory experiments [4,11,14,44,45]. In a recent work it was shown that under specific experimental conditions amino acids and nucleotide bases can be formed by gas phase chemistry [47]. Possible formation pathways were not discussed [47], but it was highlighted that gas phase mechanisms for nucleotide base formation may open a new perspective for the understanding of prebiotic chemistry. These knowledge gaps call for systematic studies aiming at establishing reliable kinetic reference data, which can be used to train photochemical models [4].

The product formation in our experiment, which is highly pressure sensitive in the low pressure range, illustrates the delicate balance of mechanistic branching in chemically activated species chemistry. In this work the mechanistic pathways can still be interpreted by analogy to combustion kinetics [28] although no detailed mechanistic scheme of the radical reactions has been provided. We think that this type of photolysis experiments lends itself for exploring the sub-mechanisms involved in high energy photon driven kinetics, which have applications both in combustion chemistry and photochemistry of planetary bodies.

## 3. Experimental

The qualitative and quantitative yields of the reaction channels were studied by an arrangement consisting of a laser flash photolysis setup (static stainless steel reactor with a volume of 5 L, excimer laser λ Physik Compex 102 (Lambda Physik, Göttingen, Germany) directly coupled to a FTIR spectrometer (IFS 66 (Bruker, Ettlingen, Germany), White optics, 4 m optical path length) for the identification and quantification of stable products. The labile species C_3_H_5_ was generated by laser-induced photodecomposition of three different precursors C_3_H_5_Cl, C_3_H_5_Br, or 1,5-hexadiene (C_6_H_10_) at 193 nm in the center of the reactor (photolysis volume ca. 10 cm × 1.7 cm^2^). The pulse energies were 40 to 80 mJ (~20 ns duration) leading to strong UV radiation. Therefore the time scale of the initiated radical chemistry (microseconds) is short compared to diffusion controlled wall loss of intermediates as tested by kinetic modeling of the experiments, see e.g., [18,48]. The quantum yields of photolysis reactions were determined for 1,5-hexadiene in agreement with [21,26] (see discussion above). For the precursors allyl bromide and allyl chloride we could not directly discriminate photolysis channels leading to C_3_H_4_ (allene, propyne) and HCl/HBr from the pathway *via* the allyl self-reaction or secondary chemistry. However, the absence of allene and propyne in co-photolysis experiments with SO_2_ [10] indicates that allyl + Br/Cl is the only significant photolysis pathway (see also discussion above). The consumption of the allyl precursors was determined, and all carbon-containing substances were quantified for a mass balance. A quantitative identification was made by the comparison with appropriately scaled pure spectra of identified compounds, leading to a total carbon mass recovery of ~40% (C_3_H_5_Cl), ~85% (C_3_H_5_Br) and ~95%–100% (1,5-hexadiene). Initial precursor concentrations were chosen such that similar (within an estimated factor of two) allyl concentrations were generated during the photolysis. Further details are given below and in [18,48].

### Chemicals

Chemicals were used without further purification: He (≥99.995%), Ar (≥99.998%), C_2_H_4_ (≥99.9%), all Messer-Griesheim (Frankfurt, Germany); C_3_H_5_Cl (≥99%), C_3_H_5_Br (≥99%), 1,5-hexadiene (≥95%), Fluka (Steinheim, Germany; now Sigma-Aldrich); C_2_H_2_ (99.5%), CH_3_CCH (99%), CH_2_CCH_2_ (≥97.5%); C_3_H_6_ (≥99.95%), Linde (München, Germany).

## 4. Conclusions

The product formation in the allyl radical self-reaction was examined at room temperature in laser flash photolysis experiments using the precursors C_3_H_5_Br, C_3_H_5_Cl, and 1,5-hexadiene (C_6_H_10_). At low pressure for all precursors an end product spectrum was found which is similar to a recent pyrolysis study, featuring high yields of propene, allene and propyne in addition to the recombination product 1,5-hexadiene. The experimental findings at low pressure were explained by specific reaction kinetics of photochemically activated allyl radicals. Pressure reduction in the C_3_H_5_Br experiments was found to induce similar trends in the product spectrum as temperature increase the pyrolysis experiment [28]. These findings exemplify the specificities of reaction kinetics involving chemically activated species, which show both, similarities and differences to established mechanisms in combustion kinetics or atmospheric chemistry. This issue is of importance for the atmospheric oxidation of alkynes [49] and for reactive particle formation processes. In the latter field we have to deal with a complex, yet only poorly understood chemistry, involving reactions of chemically activated species, which exhibits a huge influence on the formation rate and physical-chemical properties of complex particulate matter [14,15,47].
